# Pain-centered symptom-language co-occurrence networks differ between interstitial cystitis/bladder pain syndrome and fibromyalgia in Reddit patient discourse

**DOI:** 10.1038/s41598-026-58586-9

**Published:** 2026-06-22

**Authors:** Nobuo Okui, Kenta Ichino, Hisamitu Ide, Kazuhito Matsushita, Yoshihiro Ikehata, Machiko Okui, Shigeo Horie

**Affiliations:** 1 Innovative Longevity, Medicine, Juntendo University, Tokyo, 113-8421 Japan; 2 Urology, Yokosuka Urogynecology and Urology Clinic, Ootaki 2-6, Yokosuka, 238-0008 Kanagawa Japan; 3 Mathematics, Kanagawa Dental University, Inaoka-cyou 82, Yokosuka, 238-0008 Kanagawa Japan; 4 Urology, Graduate School of Medicine, Juntendo University, Tokyo, 113- 8421 Japan

**Keywords:** Diseases, Medical research, Signs and symptoms, Urology

## Abstract

Interstitial cystitis/bladder pain syndrome (IC/BPS) and fibromyalgia (FM) are chronic pain conditions frequently discussed within shared conceptual frameworks such as central sensitization. Despite this conceptual overlap, IC/BPS is clinically characterized by pelvic and urinary symptoms, whereas FM is associated with widespread pain, fatigue, sleep disturbance, and other systemic manifestations. Whether these differences are reflected in the organization of symptom-related language within patient-generated discourse remains insufficiently characterized. Patient-generated text related to IC/BPS and FM was collected from disease-specific Reddit communities using identical retrieval procedures, yielding temporally comparable corpora. A predefined evaluative lexicon was constructed from validated clinical questionnaires together with pain-related, affective, and anatomical vocabulary. Symptom-language co-occurrence networks were analyzed using a three-step framework examining global pain-centered network organization, dense-core topology and community structure, and local pain-centered lexical configurations. Both conditions exhibited pain-centered symptom-language networks. However, the FM network showed a greater concentration of symptom-language terms within the immediate network neighborhood of “pain,” whereas the IC/BPS network retained a larger peripheral structure. Dense-core analysis revealed distinct topological organization. The IC/BPS dense core retained partial modular differentiation, whereas the FM dense core formed a highly interconnected structure with minimal residual community organization. At the local level, pain-centered lexical neighborhoods also differed between conditions. In IC/BPS, “pain” was preferentially associated with pelvic, urinary, and sensory terms, whereas in FM it was more frequently associated with whole-body, fatigue-, sleep-, chronicity-, and affect-related vocabulary. These findings indicate that two chronic pain conditions sharing substantial conceptual overlap nevertheless exhibit distinct pain-centered symptom-language topologies within patient-generated discourse. Comparative network analysis of patient-generated language may provide a complementary framework for characterizing disease-specific patterns of symptom-language organization in online health communities.

**Supplementary Information:**

The online version contains supplementary material available at https://doi.org/10.1038/s41598-026-58586-9.

## Introduction

Interstitial cystitis/bladder pain syndrome (IC/BPS) and fibromyalgia (FM) are chronic pain conditions that are frequently discussed within overlapping conceptual frameworks, including central sensitization and multisystem symptom amplification^1,2^. Both conditions are characterized by persistent pain, heterogeneous symptom profiles, and the absence of definitive objective biomarkers^3,4^. Clinically, however, IC/BPS is anchored to pelvic and lower urinary tract symptoms^5,6^, whereas FM is described as a systemic syndrome involving widespread pain, fatigue, sleep disturbance, and affective symptoms^7,8^. How such differences in clinical framing are reflected in patterns of symptom-related language co-occurrence within patient-generated text remains insufficiently understood.

Most existing studies examine these conditions using predefined symptom lists or questionnaire-based assessments^9,10^. While essential for clinical evaluation, such approaches provide limited insight into how symptom-related terminology is expressed within natural language discourse^11,12^. Consequently, it remains unclear how symptom-related vocabulary as a whole—including both questionnaire-derived terms and patient-generated language—is distributed and co-occurs within patient-generated discourse^13,14^.

Beyond overall vocabulary distribution, the structure of highly central symptom-related language remains insufficiently characterized^15,16^. Central terms may form differentiated communities or aggregate into more homogeneous core structures, yet the organization of these central regions within symptom-language co-occurrence networks remains largely unknown^17,18^.

At a local level, the language surrounding the term “pain” warrants closer examination^19,20^. Rather than treating pain as a uniform construct, it remains unclear which symptom-related terms most frequently co-occur with pain and how such local co-occurrence patterns differ across patient-generated discourse^21–23^.

Patient-generated text from online peer-support communities provides an opportunity to address these questions. Network analysis of natural language permits examination of symptom-language co-occurrence patterns across global, central, and local structural scales^24,25^.

Despite their frequent conceptual linkage, it remains unclear whether IC/BPS and FM exhibit similar or distinct pain-centered language organization within patient-generated discourse. To address this question, a three-step network-analytic framework was applied to patient-generated text related to IC/BPS and FM. Step 1 examined the distribution of questionnaire-derived terms and patient-generated evaluative language within disease-specific symptom-language co-occurrence networks. Step 2 examined the dense-core structure and community organization of highly central symptom-language terms. Step 3 evaluated the local network structure surrounding the term “pain” through analyses of first-order neighbors and pain-centered triadic motifs.

## Results

### Step 1: Global pain-centered organization of symptom vocabularies

For IC/BPS, the network was constructed from 245 original posts and 4,697 associated comment threads, comprising 525,465 words. For FM, the network was derived from 752 posts and 13,062 associated comment threads, comprising 1,073,952 words.

**Fig. 1:**
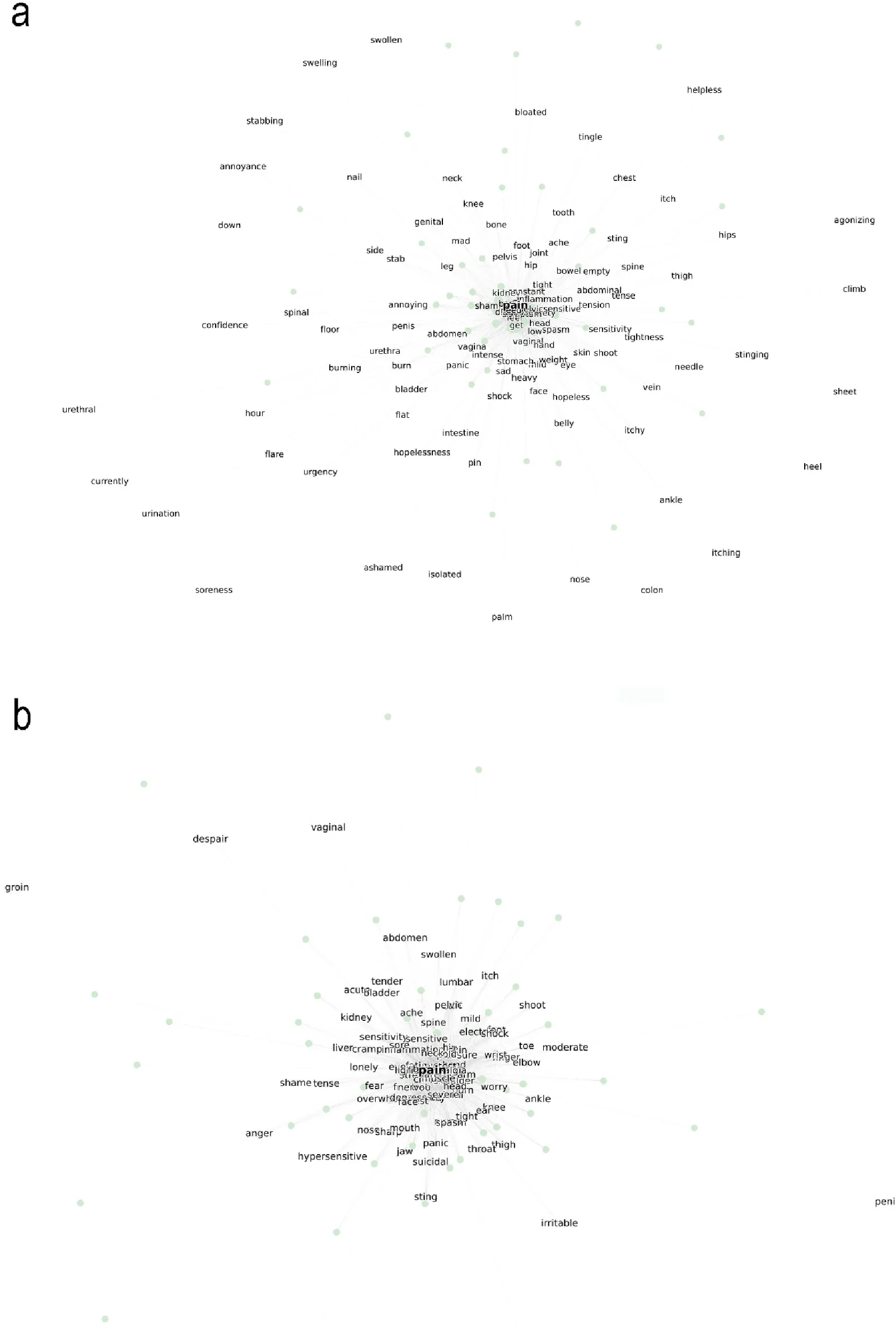
Global pain-centered symptom co-occurrence networks. (**a**) Interstitial cystitis/bladder pain syndrome (IC/BPS) network. (**b**) Fibromyalgia (FM) network. Nodes represent evaluative terms derived from clinical questionnaires, pain-related vocabulary, affective vocabulary, and anatomical vocabulary. Edges represent symptom term co-occurrence.

Figure 1a shows the IC/BPS symptom-language co-occurrence network, consisting of 78 nodes and 513 edges. All nodes belonged to a single connected component containing “pain”. Based on geodesic distance from “pain”, 68 nodes (87.18%) were located within the core (distance 0–1), whereas 10 nodes (12.82%) occupied the periphery (distance ≥ 2).

Figure 1b shows the FM symptom-language co-occurrence network, consisting of 144 nodes and 2,384 edges. All nodes likewise belonged to a single connected component containing “pain”. Under the same definition, all 144 nodes were located within the core, and no peripheral nodes were identified.

Pain-centered organization was observed in both disease-specific symptom-language networks. The FM network showed complete concentration of nodes within the pain core, whereas IC/BPS retained a peripheral structure comprising 12.82% of nodes.

The difference remained after matched-corpus subsampling, permutation-based null-model analysis, exclusion of disease-specific questionnaire vocabulary, and variation of co-occurrence window size. Higher pain-core proportions were consistently observed in FM across all robustness analyses (Supplementary Note 1; Supplementary Fig. S1–S4; Supplementary Table S1-S5).

Pain therefore occupied the position of a global organizing hub in both conditions, while symptom-language organization in FM remained more strongly concentrated around pain than in IC/BPS.

### Step 2: Dense core topology

**Fig. 2:**
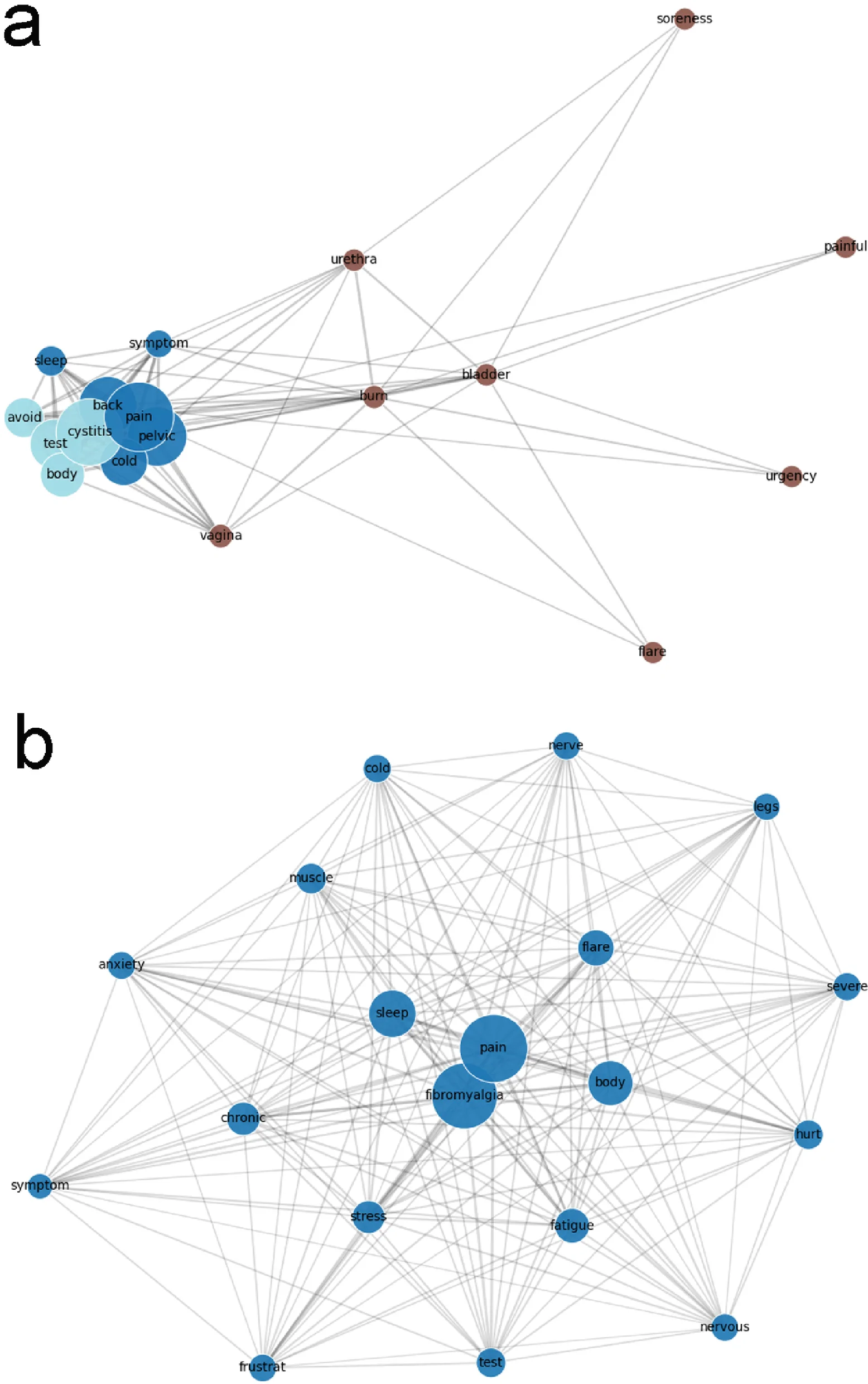
Dense core structure and community organization of symptom language in IC/BPS and FM. Nodes represent symptom-related terms. Edges represent frequent co-occurrence relationships within the dense core. Node size is proportional to weighted eigenvector centrality. Dense-core extraction was performed using the top 12% weighted eigenvector-centrality nodes followed by k-core filtering. Community assignment was performed using weighted Louvain clustering (γ = 1.0). (**a**) IC/BPS. Dense core consisting of 10 nodes and 43 edges (density = 0.956; modularity Q = 0.095). Two residual communities were identified. Community 1 included pain, pelvic, back, cold, and sleep. Community 2 included cystitis, test, body, avoid, and shame. (**b**) FM. Dense core consisting of 18 nodes and 153 edges (density = 1.000; modularity Q = 0.001). The dense core formed a fully connected network with minimal residual community structure.

Figure 2a shows the dense core of the IC/BPS network. The IC/BPS dense core consisted of 10 nodes and 43 edges (density = 0.956), indicating a highly interconnected central structure while retaining limited residual modular organization.

Figure 2b shows the corresponding dense core of the FM network. The FM dense core consisted of 18 nodes and 153 edges and formed a fully connected graph (density = 1.000), indicating complete connectivity among the most central symptom-related terms.

Both conditions exhibited highly interconnected dense cores; however, marked differences in topological organization were observed. The FM dense core displayed complete saturation, whereas the IC/BPS dense core retained a small degree of internal differentiation.

The average degree of the FM dense core (17.0) exceeded that of the IC/BPS dense core (8.6), reflecting near-maximal connectivity among central symptom terms in FM. Weighted Louvain analysis yielded a modularity value of Q = 0.095 for IC/BPS and Q = 0.001 for FM (Supplementary Table S6), indicating weak residual community structure in IC/BPS and minimal modular organization within the fully connected FM dense core.

Sensitivity analyses across alternative eigenvector-centrality thresholds and k-core parameters yielded consistent topological patterns (Supplementary Table S7). Complete or near-complete dense-core connectivity remained evident in FM, whereas partial modular differentiation persisted in IC/BPS.

### Community structure within dense cores

Figure 2a illustrates the community organization of the IC/BPS dense core. Weighted Louvain analysis identified two residual communities (Q = 0.095). The most central community, defined by cumulative eigenvector centrality, was centered on “pain” and included terms such as “symptom”, “pelvic”, “feel”, and “sleep”. This community occupied the geometric center of the dense core. The remaining communities corresponded to a urinary–sensory cluster characterized by terms such as “bladder”, “urethra”, “urgency”, and “burning”, and to a broader health-evaluative cluster including “health”, “patient”, and “avoid”. As visualized in Fig. 2a, IC/BPS symptom language retained a partially differentiated dense-core structure organized around a dominant pain-centered cluster.

In contrast, the FM dense core exhibited minimal modular organization (Q = 0.001). Although weak algorithmic partitions were detected by weighted Louvain clustering, the network formed a fully connected dense core (density = 1.000) with extensive connectivity among central symptom-related terms. Highly connected terms included “pain”, “feel”, “body”, “fatigue”, “sleep”, and common experiential descriptors. Rather than distinct community subdivision, Fig. 2b was characterized by dense integration of symptom, bodily, and experiential language within a saturated dense-core topology.

### Contrasting organizational principles between IC/BPS and FM

Taken together, Fig. 2a and b demonstrate contrasting dense-core topologies between the two conditions. In IC/BPS, “pain” occupied the most central position within a dense core that retained partial modular organization. Weighted Louvain analysis identified two communities (Q = 0.095), indicating residual structural differentiation among highly connected symptom-related terms.

In contrast, the FM dense core formed a fully connected network (density = 1.000) with negligible modularity (Q = 0.001). Although “pain” remained one of the most central nodes, the dense core exhibited extensive connectivity among central symptom-related terms and did not show meaningful community subdivision under weighted Louvain clustering.

These findings indicate that pain-centered language occupied the dense-core region of both networks, but that their internal topologies differed substantially. Whereas the IC/BPS dense core retained partial structural differentiation, the FM dense core was characterized by a highly integrated and saturated topology. Similar patterns were observed across alternative eigenvector-centrality thresholds and k-core parameters (Supplementary Tables S6–S7).

### Step 3: Pain-centered triadic motifs and first-order neighbors

Table 1 summarizes the highest-ranking pain-centered lexical triads in the IC/BPS and FM dense cores based on triangle share score.

In IC/BPS, the highest-ranking triads frequently included “pelvic”, “cystitis”, “back”, “test”, “cold”, “body”, and “avoid” in combination with “pain”. The highest-ranking triad was “pain”–“pelvic”–“cystitis”, followed by “pain”–“back”–“cystitis” and “pain”–“back”–“pelvic”.

In FM, the highest-ranking triads frequently included “fibromyalgia”, “sleep”, “body”, “flare”, “fatigue”, “chronic”, “stress”, “muscle”, “test”, and “hurt”. The highest-ranking triad was “pain”–“sleep”–“fibromyalgia”, followed by “pain”–“fibromyalgia”–“body” and “pain”–“fibromyalgia”–“flare”.

To evaluate the influence of disease-label anchoring, additional analyses were performed after removal of disease-label nodes (IC/BPS: “cystitis”; FM: “fibromyalgia”). Pain-centered triads remained identifiable in both conditions after disease-label exclusion. The number of pain-centered triads decreased from 34 to 26 in IC/BPS and from 136 to 120 in FM. Following disease-label exclusion, the highest-ranking triads were “pain”–“back”–“pelvic” in IC/BPS and “pain”–“sleep”–“body” in FM (Supplementary Table S8).

Final dense-core node composition and extraction-workflow sensitivity analyses are presented in Supplementary Tables S9 and S10.

**Table 1:** Pain-centered lexical triads in IC/BPS and FM dense cores. Highest-ranking pain-centered lexical triads (“pain”–a–b) within the dense-core networks of interstitial cystitis/bladder pain syndrome (IC/BPS) and fibromyalgia (FM). Rank: ordering by triangle share score. Triangle share score: relative contribution of each triad to all pain-centered triangles within the corresponding dense core.

Condition	Rank	Core triad (“pain”–a–b)	Triangle share score	
0	IC/BPS	1	“pain”–“pelvic”–“cystitis”	0.062
1	IC/BPS	2	“pain”–“back”–“cystitis”	0.057
2	IC/BPS	3	“pain”–“back”–“pelvic”	0.054
3	IC/BPS	4	“pain”–“test”–“cystitis”	0.049
4	IC/BPS	5	“pain”–“pelvic”–“cold”	0.046
5	IC/BPS	6	“pain”–“cystitis”–“cold”	0.042
6	IC/BPS	7	“pain”–“cystitis”–“body”	0.042
7	IC/BPS	8	“pain”–“back”–“cold”	0.040
8	IC/BPS	9	“pain”–“pelvic”–“test”	0.039
9	IC/BPS	10	“pain”–“avoid”–“cystitis”	0.038
10	FM	1	“pain”–“sleep”–“fibromyalgia”	0.030
11	FM	2	“pain”–“fibromyalgia”–“body”	0.029
12	FM	3	“pain”–“fibromyalgia”–“flare”	0.025
13	FM	4	“pain”–“fatigue”–“fibromyalgia”	0.024
14	FM	5	“pain”–“chronic”–“fibromyalgia”	0.024
15	FM	6	“pain”–“fibromyalgia”–“stress”	0.023
16	FM	7	“pain”–“fibromyalgia”–“muscle”	0.022
17	FM	8	“pain”–“fibromyalgia”–“test”	0.022
18	FM	9	“pain”–“fibromyalgia”–“hurt”	0.022

### Differential first-order lexical neighbors of “pain”

The first-order lexical neighborhood of “pain” differed between the IC/BPS and FM networks (Table 2).

In the IC/BPS network, terms including “pelvic”, “urethra”, “kidney”, “vaginal”, and “urinate” showed higher normalized co-occurrence with “pain”. In the FM network, terms including “fatigue”, “chronic”, “stress”, “muscle”, “nerve”, and “depression” showed higher normalized co-occurrence with “pain”.

Disease-specific enrichment was evaluated using corpus-size-normalized rates per million words and log2 rate ratios rather than raw co-occurrence differences.

A strict sensitivity analysis excluding disease-label terms, generic discourse terms, and ambiguous terms produced similar directional patterns (Supplementary Table S11).

Differences in the local lexical environment surrounding “pain” across the two disease-associated networks.

**Table 2:** Differential first-order lexical neighbors of “pain” in IC/BPS and FM. First-order lexical neighbors of “pain” ranked by corpus-size-normalized enrichment. Disease enrichment by rates per million words and log2 rate ratios. Positive log2 values: FM-enriched co-occurrence with “pain”. Negative log2 values: IC/BPS-enriched co-occurrence with “pain”. Raw edge weights for reference.

direction	term	w_FM	w_IC	rate_FM_per_million	rate_IC_per_million	delta_rate_per_million	log2_rate_ratio_FM_vs_IC	
0	FM-enriched	fibromyalgia	8666	0	8069.26	0.00	8069.26	13.978
1	FM-enriched	fatigue	1393	0	1297.08	0.00	1297.08	11.342
2	FM-enriched	chronic	1257	0	1170.44	0.00	1170.44	11.193
3	FM-enriched	stress	1184	0	1102.47	0.00	1102.47	11.107
4	FM-enriched	muscle	1140	0	1061.50	0.00	1061.50	11.053
5	FM-enriched	hurt	905	0	842.68	0.00	842.68	10.720
6	FM-enriched	nerve	801	0	745.84	0.00	745.84	10.544
7	FM-enriched	severe	674	0	627.59	0.00	627.59	10.295
8	FM-enriched	depression	617	0	574.51	0.00	574.51	10.167
9	FM-enriched	nervous	607	0	565.20	0.00	565.20	10.144
10	IC/BPS-enriched	back	0	1740	0.00	3311.35	-3311.35	-12.693
11	IC/BPS-enriched	cystitis	9	1887	8.38	3591.11	-3582.72	-8.660
12	IC/BPS-enriched	joint	0	82	0.00	156.05	-156.05	-8.291
13	IC/BPS-enriched	vagina	0	65	0.00	123.70	-123.70	-7.957
14	IC/BPS-enriched	penis	0	29	0.00	55.19	-55.19	-6.799
15	IC/BPS-enriched	vaginal	17	238	15.83	452.93	-437.10	-4.795
16	IC/BPS-enriched	urinate	0	6	0.00	11.42	-11.42	-4.575
17	IC/BPS-enriched	pelvic	205	2198	190.88	4182.96	-3992.08	-4.450
18	IC/BPS-enriched	urethra	5	32	4.66	60.90	-56.24	-3.574

In conclusion, although IC/BPS and FM are frequently discussed within shared chronic pain frameworks, their symptom-language networks exhibited distinct pain-centered topological organization across global, dense-core, and local structural scales. Pain occupied a central position in both conditions, yet differed in its surrounding network architecture, dense-core integration, and local lexical configurations. These findings suggest that patient-generated discourse contains disease-specific organizational patterns that are not fully captured by symptom inventories alone. Network-based analysis of patient language may therefore provide a complementary framework for investigating how symptom-related language is structured across chronic pain conditions.

## Discussion

This study examined symptom-language co-occurrence structure in IC/BPS and FM using a multi-scale network-analytic framework applied to patient-generated text^26^. Rather than focusing on symptom frequency or severity, the analysis addressed how symptom-related vocabulary is distributed, organized, and locally arranged within natural language^27–29^. Across the three analytical stages, condition-specific differences were observed in the structure of symptom-language co-occurrence networks. These findings are consistent with previous work showing that patient-generated pain discourse can be represented as network structures, that chronic pain narratives contain identifiable linguistic organization, and that open-ended patient language can be transformed into quantifiable representations of symptom-related experience^22,30,31^.

First, at the global level, the distribution of symptom-related vocabulary reflects the breadth of symptom-related language within patient discourse^16^. The observed network configurations indicate that symptom language in IC/BPS and FM is not organized solely around shared pain-related concepts, but instead occupies distinct positions within disease-specific linguistic landscapes. This suggests that questionnaire-derived terminology and patient-generated expressions coexist and co-occur differently across the two disease-specific corpora^27,30–33^. Previous studies have shown that chronic pain narratives contain identifiable linguistic structure^30,31^ and that computational analyses can capture differences in how patients describe their symptoms and everyday experiences^34^. These findings are consistent with the present network structures and suggest that global differences in symptom-language organization may influence how patients frame, prioritize, and narrate their conditions, potentially shaping communication styles and symptom reporting during clinical encounters.

Second, focusing on the central regions of the networks provides further insight into how highly salient symptom-related language is organized. The structure of dense cores indicates that central symptom-language terms do not simply cluster by prominence, but are arranged according to condition-specific network patterns. In IC/BPS, weak residual modular differentiation remained detectable within the dense core.^17,35,36^. In contrast, a more aggregated core structure in FM suggests broader integration of central symptom-related language. Previous network studies have emphasized that central nodes and bridge structures provide information beyond simple frequency counts, while also requiring careful interpretation of what centrality represents within a given network context^17,37,38^. These differences highlight that centrality alone does not capture the organizational logic of symptom discourse; rather, the internal structure of central regions reflects how symptom-related terms are grouped within each disease-specific corpus^17,37,38^.

Third, at a local level, examination of pain-centered structures shows how the term “pain” is embedded within its immediate linguistic neighborhood. The local embedding of “pain” differed between conditions, with distinct sets of neighboring symptom-related terms and triadic configurations^26,30,39^. Previous qualitative research has shown that patients actively construct and communicate pain through condition-specific narratives in order to make their symptoms understandable and credible within clinical encounters40. Such differences in local linguistic context may contribute to variation in pain descriptions, patient narratives, and shared understanding between patients and clinicians, even when pain intensity or chronicity appears similar^21,33,40^.

Last, beyond interpretation of the present findings, this framework illustrates the potential of network-based analysis of patient language as a complementary approach to traditional clinical assessment^30^. Applying similar methods across additional conditions may enable systematic comparison of symptom-language organization across disease categories^33^. Recent studies have further demonstrated that patient-generated narratives can be transformed into quantitative measures that capture clinically relevant aspects of pain experience^30^. Future work may also benefit from integrating symptom-language networks with validated approaches for extracting patient-reported symptoms from narrative text^41^. Longitudinal extensions could further clarify how symptom organization evolves over time or in response to treatment^42,43^, while integration with clinical outcomes and established symptom assessment instruments^44–48^ may reveal associations between linguistic structure, therapeutic response, and quality of life^26^.

There are several limitations that should be acknowledged. The analysis relied on self-identified patient discourse from online peer-support communities, without independent diagnostic verification^11^. Participation in such platforms may not be representative of the broader patient population, and discourse may be influenced by platform-specific norms^12^. The cross-sectional design does not capture temporal dynamics^42,43^, and lexicon-based network construction necessarily constrains analysis to predefined vocabulary^27,41^. As a co-occurrence network analysis based on a five-token sliding window, the present findings describe discourse-level patterns of lexical proximity rather than patient-level symptom networks or perceived symptom associations^15,17,37^. These factors should be considered when interpreting the findings and in designing future studies.

## Methods

### Data source and corpus construction

Patient-generated health-related text was obtained from Reddit, a publicly accessible discussion platform that hosts condition-specific peer-support communities. Reddit was selected because posts and comments are organized into disease-focused subforums (subreddits), enabling systematic retrieval of topic-constrained patient discourse.

All data collection was conducted on December 31, 2025.

Due to platform-specific constraints associated with Reddit search and content indexing, the accessible corpus does not necessarily represent the complete historical archive of a subreddit. Under these retrieval conditions, both the IC/BPS and FM corpora covered approximately one year preceding data collection, providing comparable temporal coverage across conditions.

### IC/BPS corpus

Text related to IC/BPS was collected exclusively from the subreddit r/interstitialcystitis^22^. Data retrieval was restricted to this subreddit to ensure topic specificity and to minimize contamination from unrelated discussions. All publicly visible original posts and their associated comment threads available at the time of collection were included^22^.

Posts and comments were retrieved using a constrained, subreddit-anchored query strategy. Duplicate entries arising from overlapping retrieval processes were programmatically identified and removed prior to analysis. No attempt was made to identify, track, or infer individual users across posts or comments, and all text was treated as anonymized at the time of data acquisition.

The study was designed as a corpus-level analysis of publicly available patient-generated discourse rather than a user-level investigation. Accordingly, documents were analyzed as textual units without linkage across individual accounts.

### FM corpus

FM-related patient discourse was collected using an identical data collection protocol. All text was retrieved exclusively from the subreddit r/fibromyalgia, including all publicly visible posts and their associated comments available at the time of collection^22^.

The same subreddit-constrained query strategy, duplicate-removal procedure, and anonymization policy applied to the IC/BPS corpus were used without modification. No attempt was made to identify, track, or infer individual users across posts or comments. This ensured methodological consistency across disease-specific corpora and enabled direct comparative analysis between conditions.

As in the IC/BPS corpus, analyses were performed at the corpus level using publicly available text, and documents were treated as independent textual units without linkage across user accounts.

### Temporal normalization and document selection criteria

Reddit displays posting times using relative temporal labels rather than absolute timestamps. No explicit temporal normalization, date conversion, or retrospective windowing was applied at the document level. Instead, the effective temporal coverage of each corpus was determined by platform-specific retrieval constraints inherent to Reddit’s search and content access mechanisms. Under these constraints, both the IC/BPS and FM corpora yielded posts and associated comment threads spanning approximately one year prior to the data acquisition date.

Because both disease-specific corpora were collected using the same retrieval strategy and were subject to identical platform constraints, their effective temporal coverage was comparable across conditions. Accordingly, no additional temporal filtering or investigator-defined temporal window was imposed.

Documents were eligible for inclusion if they were publicly accessible within the predefined disease-specific subreddits at the time of collection and contained non-empty text suitable for computational linguistic analysis. The analysis was conducted at the corpus level rather than at the user level. Individual users were not identified, tracked, or linked across posts or comments, and repeated contributions by the same user could not be evaluated.

Duplicate entries generated during data retrieval were removed prior to corpus construction. No additional filtering for cross-posted submissions was performed. Posts and comments were analyzed as available in the archived corpus obtained at the time of collection, and no independent verification of author identity, moderator status, bot status, or deletion history was undertaken.

Subsequent preprocessing focused on linguistic normalization. URLs, Reddit interface terms, relative-time markers, and other non-informative metadata-like tokens were removed. Disease-specific multiword expressions were normalized before tokenization, and malformed extraction artifacts were corrected or excluded using predefined rule-based procedures.

Data collection was performed manually by reviewing publicly accessible subreddit pages and copying eligible posts and associated comment threads into the study corpus. Duplicate retrieval was minimized during corpus construction by tracking previously reviewed discussion pages and avoiding repeated collection of the same publicly visible content.

### Corpus construction (Document → text)

To standardize disease-specific textual data into an analyzable format, documents in DOCX format were processed by extracting all paragraph text and table cell contents and converting them into plain text. Text extraction was performed using a scripted document parsing procedure that uniformly captured narrative text and tabular entries. The resulting plain-text files were generated separately for each disease corpus and served as the sole input for subsequent analyses.

At this stage, no natural language processing, tokenization, or network analysis was performed; this step was limited exclusively to the preparation of raw textual material for downstream computational analysis.

### Definition of the evaluative lexicon

To predefine the clinical and symptom-oriented scope of analysis and to minimize analytical arbitrariness, an evaluative lexicon was constructed prior to network analysis. This lexicon consisted of two complementary components.

First, questionnaire-derived language was obtained directly from the full text of validated clinical questionnaires for FM and IC/BPS. All questionnaire items were incorporated in their entirety, without selection or modification, to ensure comprehensive coverage of clinically defined symptom domains.

Second, supplementary cross-disease vocabularies were incorporated to represent core evaluative dimensions shared across chronic pain conditions, including pain-related terminology, affective and emotional expressions, and general anatomical references.

During lexicon construction, all text underwent systematic normalization and rule-based lemmatization to reduce superficial lexical variation. Function words and general terms not relevant to symptom evaluation were excluded using a predefined exclusion list. The final evaluative lexicon was defined as the union of questionnaire-derived vocabulary and the supplementary pain, affective, and anatomical lexicons. All subsequent co-occurrence network construction and comparative analyses were strictly restricted to terms contained within this predefined evaluative lexicon.

The affective and emotional vocabulary (AFFECT_LEX) was incorporated as a predefined exploratory lexical resource for capturing affect-related expressions in patient-generated discourse. It was not intended to function as a psychometric instrument, diagnostic scale, or quantitative measure of affective symptom severity.

### Questionnaire-based evaluative vocabulary

To define the evaluative vocabulary in a reproducible and non-arbitrary manner, this study incorporated the full text of six publicly available, validated clinical questionnaires without item-level selection or abbreviation. For FM, all items from the Revised FM Impact Questionnaire (FIQR)^43^, the FM Rapid Screening Tool (FiRST)^44^, and the 2016 American College of Rheumatology Fibromyalgia Survey Questionnaire (including the Widespread Pain Index and Symptom Severity Score)^45^ were included verbatim. For IC/BPS, all items from the Pelvic Pain and Urgency/Frequency questionnaire (PUF)^46^, the Interstitial Cystitis Symptom Index (ICSI)^47^, and the Interstitial Cystitis Problem Index (ICPI)^48^ were included in full.

All questionnaire texts were publicly available and were incorporated in their entirety to ensure complete coverage of clinically defined symptom domains. These questionnaire items were treated as fixed reference language and were used exclusively to define the evaluative lexicon applied in subsequent co-occurrence network construction. The complete questionnaire texts are provided in the Supplementary Material.

### Pain-related vocabulary (PAIN_LEX)

To comprehensively capture expressions of pain, a pain-related vocabulary (PAIN_LEX) was defined, and all of the terms listed below were included as analytic targets. This vocabulary was designed to encompass a broad range of pain-related expressions, including pain quality, intensity, temporal characteristics, sensory abnormalities, and both everyday patient language and medically specialized terminology.

“pain”, “pains”, “painful”, “ache”, “aches”, “aching”, “ached”, “sore”, “sores”, “soreness”, “hurt”, “hurts”, “hurting”, “hurtful”, “burn”, “burns”, “burning”, “stinging”, “sting”, “stings”, “stung”, “searing”, “scalding”, “scorching”, “raw”, “tender”, “tenderness”, “sensitive”, “sensitivity”, “hypersensitive”, “hypersensitivity”, “irritated”, “irritation”, “inflamed”, “inflammation”, “agonizing”, “excruciating”, “unbearable”, “intense”, “severe”, “mild”, “moderate”, “constant”, “persistent”, “chronic”, “acute”, “flare”, “flares”, “flaring”, “flareup”, “throb”, “throbs”, “throbbing”, “pulsing”, “pulse”, “pulsatile”, “sharp”, “sharply”, “stabbing”, “stab”, “stabs”, “piercing”, “shooting”, “shoot”, “shoots”, “electric”, “electrical”, “tingle”, “tingles”, “tingling”, “pins”, “needles”, “numb”, “numbness”, “numbed”, “numbingly”, “cramp”, “cramps”, “cramping”, “spasm”, “spasms”, “spasming”, “spastic”, “tight”, “tightness”, “stiff”, “stiffness”, “rigid”, “rigidity”, “pressure”, “pressured”, “pressing”, “compressing”, “compression”, “heaviness”, “heavy”, “weight”, “weighted”, “fullness”, “bloated”, “bloating”, “swollen”, “swelling”, “puffiness”, “inflation”, “scratchy”, “itch”, “itches”, “itching”, “itchy”, “prickly”, “prickle”, “prickles”, “prickling”, “grinding”, “gnawing”, “dull”, “dully”, “dullness”, “nagging”, “annoying”, “annoyance”, “twinge”, “twinges”, “twinging”, “shock”, “shocks”, “shocking”, “allodynia”, “hyperalgesia”, “paresthesia”, “dysesthesia”, “neuropathic”, “nociceptive”.

All terms were applied after text normalization and rule-based lemmatization. Subsequent co-occurrence analyses and network construction were strictly restricted to this predefined pain-related vocabulary set.

### Affective and emotional vocabulary (AFFECT_LEX)

To comprehensively capture expressions related to anxiety, depression, and emotional responses, an affective and emotional vocabulary (AFFECT_LEX) was defined, and all of the terms listed below were included as analytic targets. This vocabulary was designed to encompass a wide range of affective states and emotional reactions, including anxiety, fear, tension, depressive mood, anger, social withdrawal, reduced motivation, ruminative thinking, diminished self-worth, and suicide-related expressions.

“anxiety”, “anxious”, “worry”, “worries”, “worried”, “worrying”, “nervous”, “nervousness”, “panic”, “panics”, “panicked”, “panicky”, “fear”, “fears”, “feared”, “fearful”, “scared”, “terrified”, “dread”, “dreaded”, “stress”, “stressed”, “stressing”, “stressful”, “tension”, “tense”, “overwhelm”, “overwhelmed”, “overwhelming”, “uneasy”, “restless”, “restlessness”, “agitated”, “agitation”, “irritated”, “irritable”, “irritability”, “frustrated”, “frustration”, “annoyed”, “annoyance”, “anger”, “angry”, “mad”, “furious”, “rage”, “resentful”, “resentment”, “guilty”, “guilt”, “ashamed”, “shame”, “embarrassed”, “embarrassment”, “sad”, “sadness”, “depressed”, “depression”, “low”, “down”, “downcast”, “blue”, “tearful”, “cry”, “crying”, “hopeless”, “hopelessness”, “helpless”, “helplessness”, “despair”, “desperate”, “empty”, “emptiness”, “lonely”, “loneliness”, “isolated”, “isolation”, “withdrawn”, “withdrawal”, “apathy”, “apathetic”, “numb”, “numbness”, “flat”, “flattened”, “unmotivated”, “motivation”, “overthinking”, “ruminate”, “rumination”, “catastrophize”, “catastrophizing”, “selfblame”, “selfhate”, “hateful”, “suicidal”, “suicide”, “worthless”, “worthlessness”, “confidence”, “insecure”, “insecurity”, “sensitive”, “hypersensitive”, “hypervigilant”, “hypervigilance”, “paranoid”, “paranoia”.

All terms were applied after text normalization and rule-based lemmatization. Subsequent co-occurrence analyses and network construction were strictly restricted to this predefined affective vocabulary set.

### Anatomical vocabulary (ANATOMY_LEX)

To comprehensively capture references to body parts and anatomical structures, an anatomical vocabulary (ANATOMY_LEX) was defined, and all of the terms listed below were included as analytic targets. This vocabulary was designed to encompass whole-body structures, the trunk, upper and lower extremities, internal organs, the genitourinary system, sensory organs, and body-part expressions commonly used in everyday patient language.

“body”, “anatomy”, “skin”, “bone”, “bones”, “muscle”, “muscles”, “joint”, “joints”, “nerve”, “nerves”, “tendon”, “tendons”, “ligament”, “ligaments”, “artery”, “arteries”, “vein”, “veins”, “heart”, “lung”, “lungs”, “chest”, “rib”, “ribs”, “diaphragm”, “abdomen”, “abdominal”, “belly”, “stomach”, “gut”, “bowel”, “intestine”, “intestines”, “colon”, “rectum”, “anus”, “pelvis”, “pelvic”, “hip”, “hips”, “groin”, “perineum”, “genital”, “genitals”, “vulva”, “vaginal”, “vagina”, “uterus”, “cervix”, “ovary”, “ovaries”, “fallopian”, “prostate”, “penis”, “scrotum”, “testicle”, “testicles”, “urethra”, “urethral”, “bladder”, “kidney”, “kidneys”, “ureter”, “ureters”, “liver”, “spleen”, “pancreas”, “gallbladder”, “throat”, “neck”, “cervical”, “shoulder”, “shoulders”, “clavicle”, “collarbone”, “scapula”, “arm”, “arms”, “upperarm”, “forearm”, “elbow”, “elbows”, “wrist”, “wrists”, “hand”, “hands”, “palm”, “palms”, “finger”, “fingers”, “thumb”, “thumbs”, “knuckle”, “knuckles”, “nail”, “nails”, “back”, “spine”, “spinal”, “vertebra”, “vertebrae”, “lumbar”, “thoracic”, “sacral”, “tailbone”, “coccyx”, “waist”, “flank”, “side”, “sides”, “buttock”, “buttocks”, “glute”, “glutes”, “leg”, “legs”, “thigh”, “thighs”, “knee”, “knees”, “shin”, “shins”, “calf”, “calves”, “ankle”, “ankles”, “foot”, “feet”, “heel”, “heels”, “toe”, “toes”, “arch”, “arches”, “sole”, “soles”, “head”, “skull”, “brain”, “forehead”, “temple”, “temples”, “face”, “cheek”, “cheeks”, “jaw”, “chin”, “mouth”, “lip”, “lips”, “tongue”, “teeth”, “tooth”, “gum”, “gums”, “nose”, “nostril”, “nostrils”, “sinus”, “sinuses”, “eye”, “eyes”, “eyelid”, “eyelids”, “ear”, “ears”, “scalp”, “hair”, “crown”, “neckline”, “trachea”, “esophagus”, “shoulderblade”, “armpit”, “axilla”, “breast”, “breasts”, “nipple”, “nipples”, “sternum”, “collar”, “upperback”, “lowerback”, “midback”.

All terms were applied after text normalization and rule-based lemmatization. Subsequent co-occurrence analyses and network construction were strictly restricted to this predefined anatomical vocabulary set.

### Step 1: Construction of disease-specific symptom co-occurrence networks

In Step 1, symptom co-occurrence networks were constructed separately for IC/BPS and FM using patient-generated text. All texts were normalized and filtered using a predefined evaluative lexicon (EvalLexicon), consisting of questionnaire-derived terms, pain-related vocabulary, affective expressions, and anatomical references.

Co-occurrence relationships were extracted using a sliding window approach (window size = 5). Sliding windows were applied to the filtered token sequence and were not constrained by sentence boundaries. Word pairs exceeding a minimum co-occurrence threshold (min_edge = 5) were represented as weighted, undirected edges, and isolated nodes were removed. The resulting networks represented disease-specific symptom-language structures.

To characterize the global organization of each network with respect to pain, geodesic distances from the node pain were computed for all nodes in the network. Nodes with a distance of 0 or 1 from pain were operationally defined as the core, whereas nodes with a distance of 2 or greater were defined as the periphery.

This distance-based definition provides a topology-driven and model-independent measure of pain-centeredness, allowing global structural comparison across disease-specific networks without relying on community assignments or local motif definitions.

Robustness analyses were performed to evaluate the stability of pain-centered organization. These analyses included matched-corpus subsampling, permutation-based null-model analysis preserving symptom-language token frequencies, shared-vocabulary-only analysis excluding disease-specific questionnaire-derived terms, and sensitivity analyses using alternative co-occurrence window sizes (3, 5, and 10 tokens).

### Step 2: Dense core extraction and community identification

To examine the internal organization of highly central symptom language, the analysis focused on the dense core of each disease-specific network derived in Step 1.

Global eigenvector centrality was computed for all nodes in each network. Nodes within the top 12% of weighted eigenvector centrality were retained to define the primary dense-core network. This procedure provided an objective topology-based method for identifying the most centrally connected symptom-language nodes without requiring investigator-defined node retention.

To evaluate the robustness of dense-core topology, additional sensitivity analyses were performed using alternative eigenvector-centrality thresholds (10%, 12%, and 15%). Dense-core topology was further evaluated using k-core filtering with k = 6, 8, and 10. Node number, edge number, network density, average degree, Louvain community number, and modularity were compared across alternative extraction settings.

The resulting dense core subgraph was then examined for internal community organization. Community labels were inherited from Louvain community detection performed on the full network, ensuring that community assignments were not recalculated within the reduced subgraph. For visualization, node size represented eigenvector centrality recomputed within the dense core, while node color indicated community membership.

Identification of the central community within each dense core was based on the cumulative sum of eigenvector centrality across nodes belonging to each community. This criterion provides a topology-driven and model-independent measure of community-level centrality. Communities containing the node “pain” were additionally identified to distinguish pain-centered modules from other highly central structures.

Sensitivity analyses were performed across alternative eigenvector-centrality thresholds (10%, 12%, and 15%) and k-core parameters (6, 8, and 10) to evaluate the robustness of dense-core topology.

### Step 3: Pain-centered triadic motifs and first-order lexical neighbors

Pain-centered local network structure was examined using two complementary analyses: triadic motif enumeration within the dense-core network and first-order lexical neighbor enrichment in the full disease-specific co-occurrence network.

For triadic motif analysis, a pain-centered triad was defined as a fully connected three-node clique consisting of “pain” and two additional terms (a, b). All such triads incident to “pain” were enumerated within the dense-core network for each condition. For each endpoint pair (a, b), a triangle share score was calculated as the relative contribution of that pair to the complete set of “pain”-centered triangles in the corresponding dense core. Community membership of the endpoint terms was annotated using Louvain community labels inherited from the full network, allowing identification of within-community and cross-community pain-centered configurations. Triads were ranked according to triangle share score.

To assess the influence of disease-label anchoring, sensitivity analyses were performed after removal of disease-label nodes before triad extraction. The excluded disease-label nodes were “cystitis” for IC/BPS and “fibromyalgia” for FM. Pain-centered triads were then recalculated using the modified dense-core networks.

Pain-centered first-order lexical neighbor analysis was performed using the full disease-specific symptom-language co-occurrence networks. In each network, the node “pain” was selected, and all directly connected first-order neighbors were identified. For each neighboring term, the edge weight between “pain” and that term was extracted separately for IC/BPS and FM. Raw edge weights were retained for reference but were not used as the primary basis for cross-corpus enrichment because the two corpora differed in size.

Disease-specific enrichment of first-order lexical neighbors was evaluated using corpus-size-normalized co-occurrence rates per million words and log2 rate ratios. For each term, normalized rates were calculated separately for the IC/BPS and FM corpora, and the log2 rate ratio was used to indicate the direction and magnitude of enrichment. Positive log2 rate ratios indicated FM-enriched co-occurrence with “pain”, whereas negative values indicated IC/BPS-enriched co-occurrence with “pain”. Terms were ranked by normalized enrichment rather than raw co-occurrence difference.

A strict sensitivity analysis was also performed after excluding disease-label terms, generic discourse terms, and ambiguous terms. First-order lexical neighbors of “pain” were then recalculated, and disease-specific enrichment was again evaluated using normalized rates per million words and log2 rate ratios.

### Statistical analysis and computational framework

All analyses were conducted using Python (Python Software Foundation, Wilmington, DE, USA) in the Google Colab environment (Google LLC, Mountain View, CA, USA). The analytical workflow was fully scripted to ensure reproducibility. Text preprocessing and corpus construction were performed using pandas, NLTK, and custom Python scripts. Network construction, graph-theoretical analyses, and topological measurements were performed using NetworkX. Community detection was conducted using the Louvain algorithm.

Robustness analyses included matched-corpus subsampling, permutation-based null-model analyses preserving symptom-language token frequencies, shared-vocabulary-only analyses, disease-label exclusion analyses, and sensitivity analyses across alternative co-occurrence window sizes, eigenvector-centrality thresholds, and k-core parameters. Empirical p-values for permutation analyses were calculated from the corresponding null distributions.

For first-order lexical neighbor analyses, disease-specific enrichment was evaluated using corpus-size-normalized co-occurrence rates per million words and log2 rate ratios.

Random seeds were fixed when stochastic procedures were applied. Programming assistance was obtained from ChatGPT (OpenAI, San Francisco, CA, USA), whereas all analyses, parameter selection, result verification, and manuscript preparation were performed by the authors.

## Supplementary Information

Below is the link to the electronic supplementary material.

Supplementary Material 1

Supplementary Material 2

## Data Availability

All patient-generated language corpora analyzed in this study are publicly available in Zenodo at https://doi.org/10.5281/zenodo.18465160.

## **Code availability**

All analysis and visualization code used in this study is publicly available on GitHub at https://github.com/Curiosity-Mars/ICvsFM/.
