# Supplementary material for: Pain-centered symptom-language co-occurrence networks differ between interstitial cystitis/bladder pain syndrome and fibromyalgia in Reddit patient discourse
Source: Sci Rep. 2026 Jun 22;16:28437. doi: 10.1038/s41598-026-58586-9 (PMC13562692; doi:10.1038/s41598-026-58586-9)
Supplement: Supplementary file 1 [file 41598_2026_58586_MOESM2_ESM.pdf]

Supplementary Information

Supplementary Text 1

Questionnaire item wording used to define the evaluative lexicon (item text only)

The following questionnaire item wordings were incorporated verbatim to define the questionnaire-based evaluative vocabulary used in this study.

Items are presented without response options, scoring scales, subtotals, or diagnostic thresholds, as questionnaire scores were not used in any analysis.

All questionnaire texts are publicly available and were included in their entirety to ensure non-arbitrary and reproducible definition of clinically grounded symptom language.

Fibromyalgia questionnaires

Revised Fibromyalgia Impact Questionnaire

(FIQR)

Domain 1: Function

1. Brush or comb your hair
2. Walk continuously for 20 minutes
3. Prepare a homemade meal
4. Vacuum, scrub, or sweep floors
5. Lift and carry a bag full of groceries
6. Climb one flight of stairs

7. Change bed sheets
8. Sit in a chair for 45 minutes
9. Go shopping for groceries

Domain 2: Overall impact

10. Fibromyalgia prevented me from accomplishing goals for the week
11. I was completely overwhelmed by my fibromyalgia symptoms

Domain 3: Symptoms

12. Please rate your level of pain
13. Please rate your level of energy
14. Please rate your level of stiffness
15. Please rate the quality of your sleep
16. Please rate your level of depression
17. Please rate your level of memory problems
18. Please rate your level of anxiety
19. Please rate your level of tenderness to touch
20. Please rate your level of balance problems
21. Please rate your level of sensitivity to loud noises, bright lights, odors, and cold

---

Fibromyalgia Rapid Screening Tool (FiRST)

1. I have pain all over my body.
2. My pain is accompanied by a continuous and very unpleasant general fatigue.
3. My pain feels like burns, electric shocks, or cramps.
4. My pain is accompanied by other unusual sensations throughout my body, such as pins and needles, tingling, or numbness.
5. My pain is accompanied by other health problems such as digestive problems, urinary problems, headaches, or restless legs.
6. My pain has a significant impact on my life, particularly on my sleep and my ability to concentrate, making me feel slower in general.

Fibromyalgia Survey Questionnaire

(2016 American College of Rheumatology criteria)

Symptom Severity Scale (SSS): symptom descriptors

- Fatigue
- Trouble thinking or remembering
- Waking up tired (unrefreshed)
- Pain or cramps in lower abdomen
- Depression
- Headache

Widespread Pain Index (WPI): body regions

Left upper region

- Left jaw
- Left shoulder girdle
- Left upper arm
- Left lower arm and/or left wrist/hand
- Left elbow

Right upper region

- Right jaw
- Right shoulder girdle
- Right upper arm
- Right lower arm and/or right wrist/hand
- Right elbow

Left lower region

- Left hip and/or left buttock
- Left upper leg and/or left groin
- Left lower leg and/or left ankle/foot

- Left knee

Right lower region

- Right hip and/or right buttock
- Right upper leg and/or right groin
- Right lower leg and/or right ankle/foot
- Right knee

Axial region

- Neck
- Upper back
- Lower back
- Chest
- Abdomen

Interstitial cystitis / bladder pain syndrome questionnaires

Interstitial Cystitis Symptom Index (ICSI)

During the past month:

1. How often have you felt the strong need to urinate with little or no warning?
2. Have you had to urinate less than 2 hours after you have finished urinating?
3. How often did you, most typically, get up at night to urinate?
4. Have you experienced pain or burning in your bladder?

Interstitial Cystitis Problem Index (ICPI)

During the past month, how much has each of the following been a problem for you:

1. Frequent urination during the day?
2. Getting up at night to urinate?
3. Need to urinate with little warning?
4. Burning pain, discomfort, or pressure in your bladder?

Pelvic Pain and Urgency/Frequency (PUF) Patient Symptom Scale

1. How many times do you go to the bathroom during the day?
2. a. How many times do you go to the bathroom at night?

   b. If you get up at night to go to the bathroom, to what extent does it bother you?

3. Are you currently sexually active?
4. a. If you are sexually active, do you now or have you ever had pain or symptoms during or after sexual activity?

   b. If you have pain, does it make you avoid sexual activity?

5. Do you have pain associated with your bladder or in your pelvis (vagina, labia, lower abdomen, urethra, or perineum)?
6. a. If you have pain, is it usually mild, moderate, or severe?

   b. Does your pain bother you?

7. Do you still have urgency shortly after you go to the bathroom?
8. a. If you have urgency, is it usually mild, moderate, or severe?

b. Does your urgency bother you?

Supplementary References

1. Bennett RM et al. *The Revised Fibromyalgia Impact Questionnaire (FIQR): validation and psychometric properties.* Arthritis Research & Therapy. 2009.
2. Perrot S et al. *Development and validation of the Fibromyalgia Rapid Screening Tool (FiRST).* Pain. 2010.
3. Wolfe F et al. *2016 Revisions to the 2010/2011 fibromyalgia diagnostic criteria.* Seminars in Arthritis and Rheumatism. 2016.
4. O'Leary MP, Sant GR et al. *The Interstitial Cystitis Symptom Index and Problem Index.* Urology. 1997.
5. Parsons CL. *Pelvic Pain and Urgency/Frequency (PUF) Patient Symptom Scale.* 2000.

Supplementary Note 1. (Step 1)

Robustness analyses of global pain-centered symptom-language organization

Because the FM corpus contained substantially more posts, comments, and retained symptom-language tokens than the IC/BPS corpus, additional analyses were performed to evaluate whether the observed difference in pain-centered aggregation could be explained by corpus-size imbalance, disease-specific vocabulary composition, or analytical parameter choices.

First, matched-corpus subsampling was performed by repeatedly downsampling the FM corpus to match the number of retained symptom-language tokens in the IC/BPS corpus (9,776 tokens). Across 100 iterations, the FM network retained a high pain-core proportion (mean 95.46%, SD 1.69%), exceeding the value observed in the original IC/BPS network (87.18%) (Supplementary Fig. S1).

Second, permutation-based null-model analyses were performed by randomly shuffling retained symptom-language tokens while preserving token frequencies. The observed FM network exhibited a higher pain-core proportion than expected under the null model (100.00% vs. 97.15%; empirical $p = 0.0297$), whereas the IC/BPS network exhibited a larger peripheral structure than expected under random token ordering (Supplementary Fig. S2).

Third, disease-specific questionnaire-derived terms were removed and the analysis was repeated using only shared pain-related, affective, and anatomical vocabulary. The resulting pain-core proportions remained higher in FM than in IC/BPS (98.25% vs. 89.23%), indicating that the observed difference was not solely attributable to disease-specific lexicon composition (Supplementary Fig. S3).

Finally, sensitivity analyses were performed using alternative co-occurrence window sizes of 3, 5, and 10 tokens. The relative ordering of the two disease-

specific networks remained unchanged across all tested parameter settings (Supplementary Fig. S4).

Collectively, these robustness analyses indicate that the stronger pain-centered aggregation observed in the FM network was preserved after corpus-size matching, permutation-based null modeling, exclusion of disease-specific questionnaire vocabulary, and variation of co-occurrence window size.

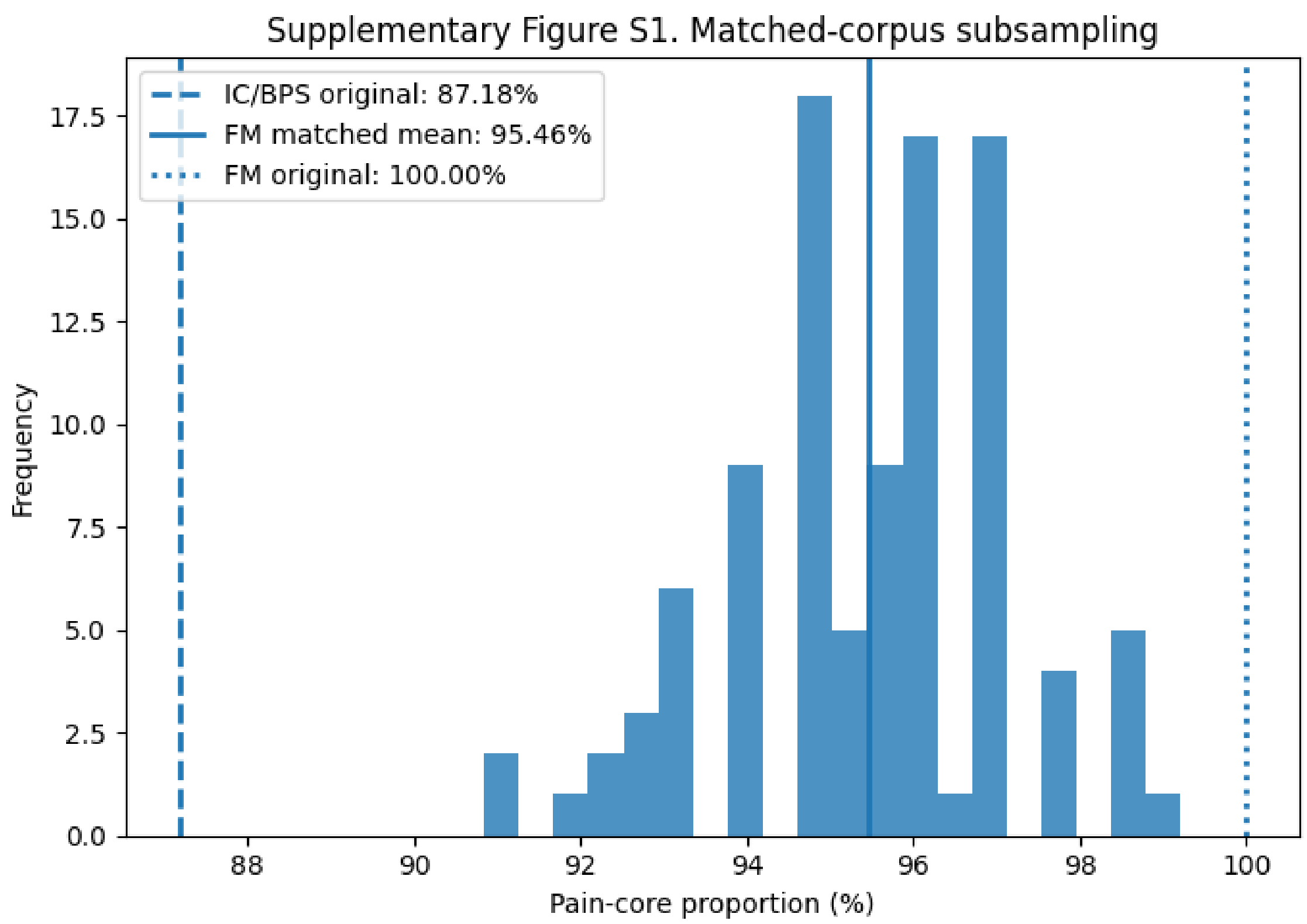

Supplementary Figure S1.Matched-corpus subsampling analysis

Distribution of pain-core proportions obtained from 100 matched-corpus subsampling iterations of the FM corpus. In each iteration, the FM corpus was randomly down sampled to match the number of retained symptom-language tokens in the IC/BPS corpus (9,776 tokens). The vertical dashed line indicates the observed IC/BPS pain-core proportion (87.18%), the solid line indicates the mean matched-corpus FM value (95.46%), and the dotted line indicates the original FM network value (100.00%). The higher pain-centered aggregation

observed in FM remained after corpus-size matching.

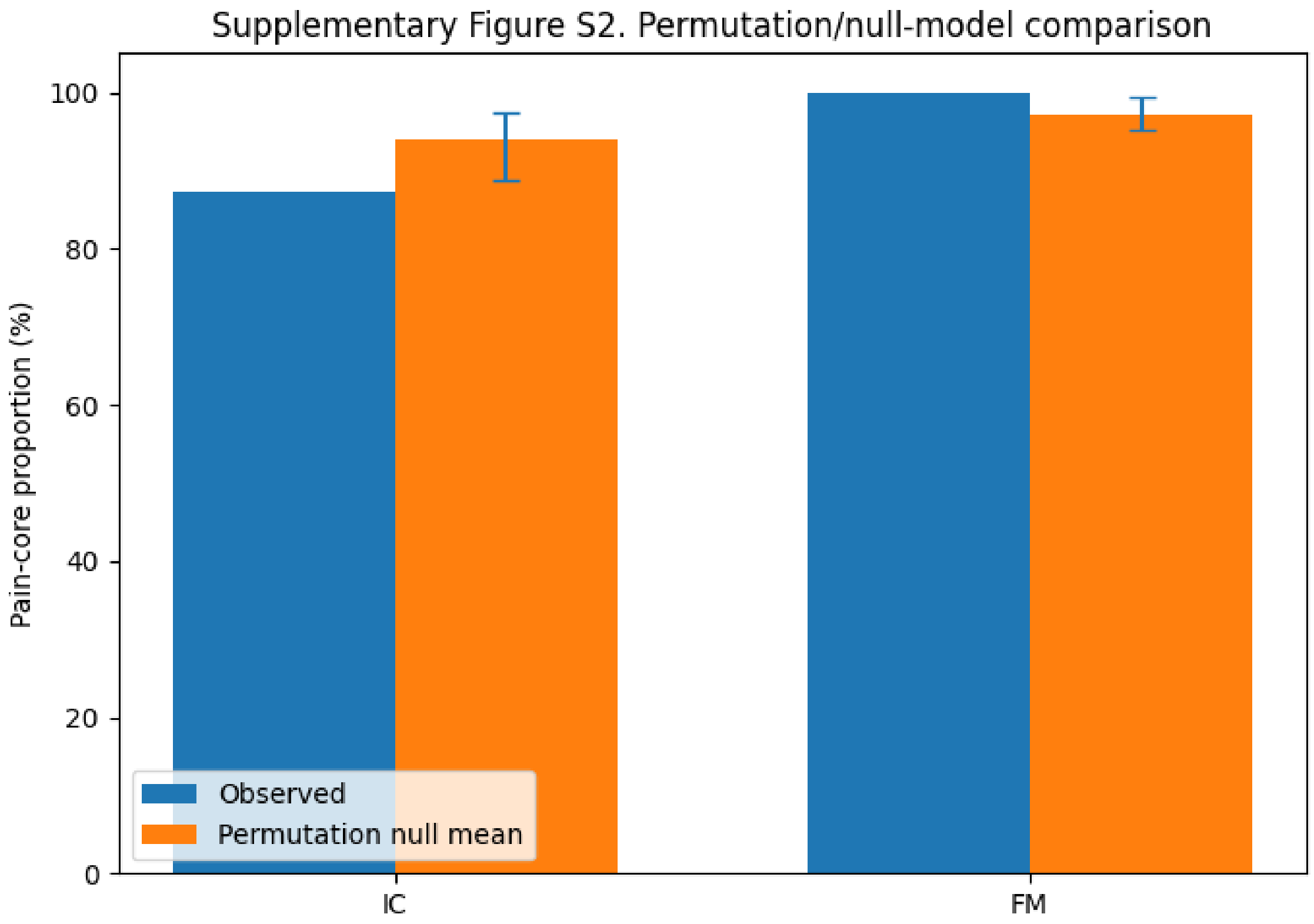

Supplementary Figure S2.Permutation-based null-model comparison

Observed pain-core proportions compared with permutation-based null models. For each condition, retained symptom-language tokens were randomly shuffled 100 times while preserving overall token frequencies. Bars indicate observed and null-model mean values; error bars represent the 2.5th–97.5th percentile range of the null distribution. The FM network exhibited a significantly higher pain-core proportion than expected under the null model (observed 100.00%, null mean 97.15%, empirical p = 0.0297), whereas the IC/BPS network exhibited a larger peripheral structure than expected under random token ordering.

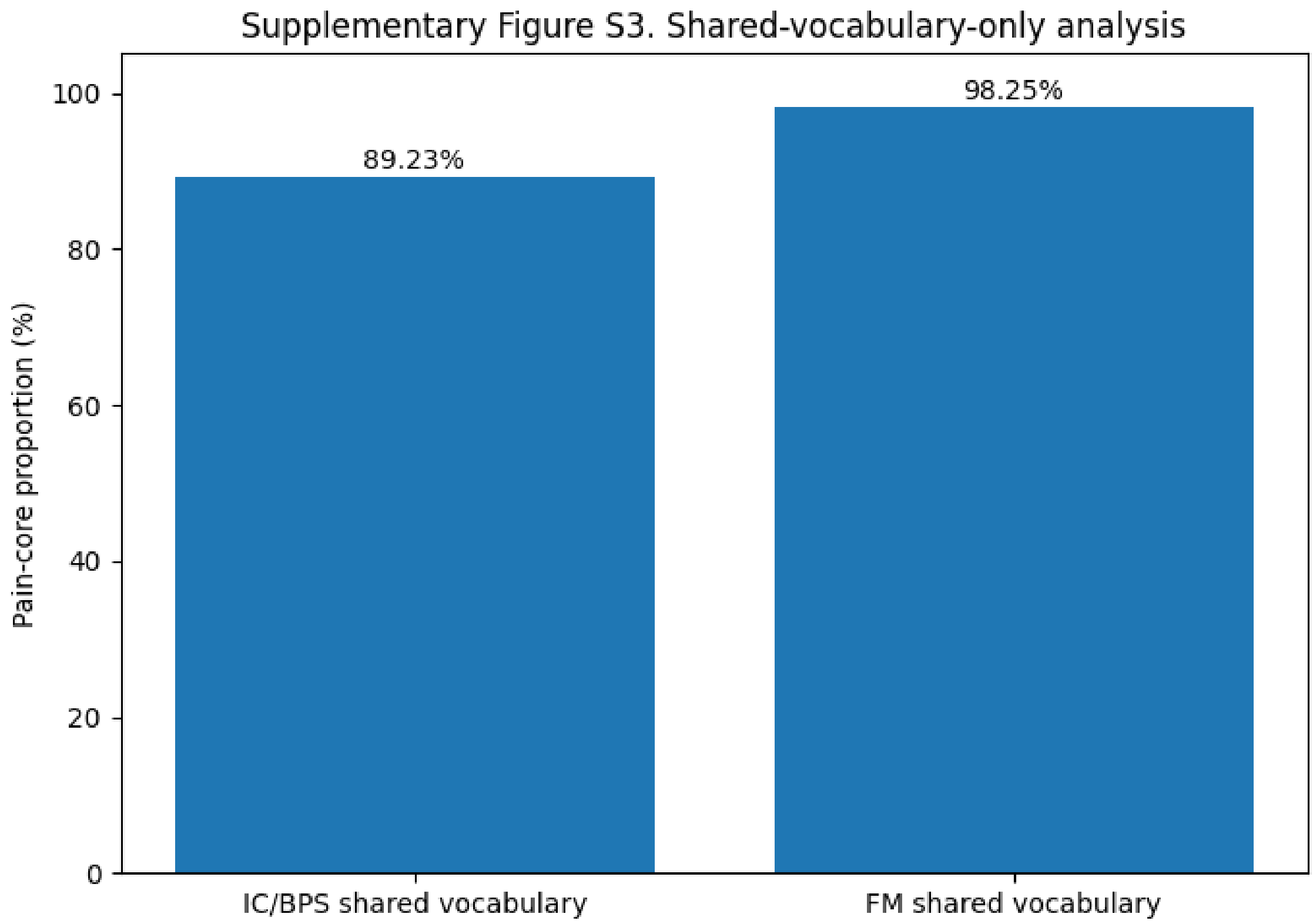

Supplementary Figure S3.Shared-vocabulary-only analysis

Pain-core proportions after excluding disease-specific questionnaire-derived vocabulary and restricting the lexicon to shared pain-related, affective, and anatomical terms. The FM network retained a substantially higher pain-core proportion than the IC/BPS network (98.25% vs. 89.23%), indicating that the observed contrast was not driven solely by disease-specific questionnaire terminology.

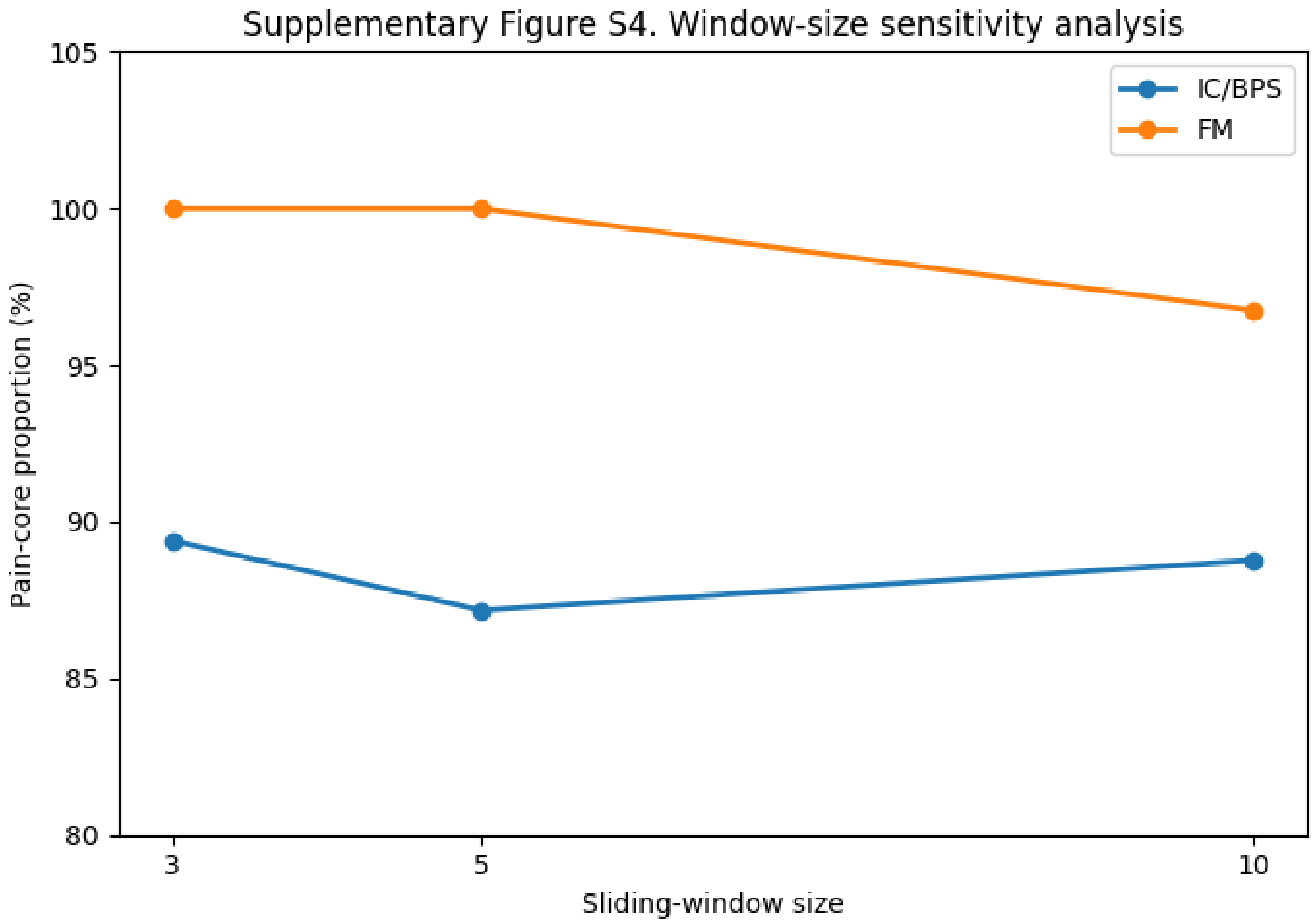

Supplementary Figure S4.Window-size sensitivity analysis

Sensitivity analysis across alternative co-occurrence window sizes. Networks were reconstructed using sliding windows of 3, 5, and 10 tokens. The relative difference between FM and IC/BPS remained stable across all tested window sizes, indicating that the observed pain-centered organization was not dependent on the specific window parameter used in the primary analysis.

**Analysis**	**IC_core_pct**	**FM_core_pct**	**Notes**	
0	Original v2 network	87.180000	100.000000	Observed one-year corpus without corpus-size m...
1	Matched-corpus subsampling	87.180000	95.460910	FM downsampled to IC retained-token count;

				mea...
2	Shared-vocabulary only	89.230769	98.245614	Disease-specific questionnaire-derived terms e...
3	Window-size sensitivity: window=3	89.393939	100.000000	Alternative sliding-window size
4	Window-size sensitivity: window=5	87.179487	100.000000	Alternative sliding-window size

Supplementary Table S1.Robustness summary for Step 1 analyses

Summary of robustness analyses for global pain-centered symptom-language organization. Pain-core proportions are reported for the original observed networks, matched-corpus subsampling, shared-vocabulary-only analysis, and alternative co-occurrence window sizes. Across all analyses, FM consistently exhibited a higher pain-core proportion than IC/BPS.

Supplementary Table S2 (Excel).Complete inventory of the evaluation lexicon used for Step 1 network construction

Complete list of all 166 terms included in the final evaluation lexicon (EvalLexicon). For each term, the source vocabulary and semantic domain are provided. Source categories include IC/BPS questionnaire-derived terms, FM questionnaire-derived terms, pain-related vocabulary, affective vocabulary, and anatomical vocabulary. This table is provided as an Excel file because of its size.

	lexicon_component	n_terms
0	IC/BPS questionnaire-derived terms	19
1	FM questionnaire-derived	50

	terms	
2	Pain-related vocabulary	46
3	Affective vocabulary	27
4	Anatomical vocabulary	53
5	Shared cross-disease vocabulary (pain + affect...	126
6	Disease-specific questionnaire vocabulary	67

Supplementary Table S3. Composition of the evaluation lexicon

Summary of vocabulary sources used for network construction. The final EvalLexicon contained 166 unique terms derived from disease-specific questionnaire vocabulary, pain-related vocabulary, affective vocabulary, and anatomical vocabulary. Shared cross-disease vocabulary consisted of pain-related, affective, and anatomical terms.

condition	stage	tokens_or_units	unique_terms	notes	
0	IC/BPS	Raw text	515031		Raw characters = 2844385
1	IC/BPS	After normalization	525794	14426	Lowercase; punctuation, hyphen, and slash norm...
2	IC/BPS	After rule-based lemmatization	525794	13146	Simple suffix handling: ing, ed, es; final s r...
3	IC/BPS	After length filter	370657	12714	Retained terms with length >= 3
4	IC/BPS	After exclusion-word removal	309177	12626	Removed function words, questionnaire

					instruct...
5	IC/BPS	After EvalLexicon filtering	9776	95	Tokens retained for co-occurrence network cons...
6	IC/BPS	Final network nodes	78	78	Nodes retained after edge-threshold filtering ...
7	IC/BPS	Final network edges	513		Weighted undirected edges retained with min_ed...
8	FM	Raw text	962311		Raw characters = 5276334
9	FM	After normalization	972052	23228	Lowercase; punctuation, hyphen, and slash norm...
10	FM	After rule-based lemmatization	972052	21120	Simple suffix handling: ing, ed, es; final s r...
11	FM	After length filter	707521	20630	Retained terms with length >= 3
12	FM	After exclusion-word removal	532841	20519	Removed function words, questionnaire instruct...

13	FM	After EvalLexicon filtering	32468	155	Tokens retained for co-occurrence network cons...
14	FM	Final network nodes	144	144	Nodes retained after edge-threshold filtering ...

Supplementary Table S4. Preprocessing audit and token-retention workflow

Token counts and unique-term counts at each preprocessing stage for the IC/BPS and FM corpora. Values are reported for raw text, normalization, rule-based lemmatization, length filtering, exclusion-word removal, EvalLexicon filtering, and final network construction. This audit provides transparency regarding token retention and network generation.

analysis_term	display_term	source	domain	IC_questionnaire	FM_questionnaire	PAIN_LEX	AFFECT_LEX	ANATOMY_LEX	shared_cross_disease	disease_specific_questionnaire		analysis_term
0	abdomen	abdomen	IC/BPS questionnaire; FM questionnai	anatomical vocabulary; disease-specific	1	1	0	0	1	1	1	0

			re; ANATOM...	questi...								
1	ach	aching	PAIN_LEX	pain-related vocabulary	0	0	1	0	0	1	0	1
2	ache	ache	PAIN_LEX	pain-related vocabulary	0	0	1	0	0	1	0	2
3	acute	acute	PAIN_LEX	pain-related vocabulary	0	0	1	0	0	1	0	3
4	agitat	agitated	AFFECT_LEX	affective vocabulary	0	0	0	1	0	1	0	4
5	anger	anger	AFFECT_LEX	affective vocabulary	0	0	0	1	0	1	0	5
6	ank	ank	ANA	anat	0	0	0	0	1	1	0	6

	le	le	TOMY_LEX	omical vocabulary								
7	anxiety	anxiety	FM questionnaire; AFFECT_LEX	affective vocabulary; disease-specific questio...	0	1	0	1	0	1	1	7
8	anxious	anxious	AFFECT_LEX	affective vocabulary	0	0	0	1	0	1	0	8
9	apathy	apathy	AFFECT_LEX	affective vocabulary	0	0	0	1	0	1	0	9
10	arm	arm	FM questionnaire; ANATOMY_LEX	anatomical vocabulary; disease-specific	0	1	0	0	1	1	1	10

				questi…								
11	avoid	avoid	IC/BPS questionnaire	disease-specific questionnaire	1	0	0	0	0	0	1	11
12	back	back	FM questionnaire; ANATOMY_LEX	anatomical vocabulary; disease-specific questi…	0	1	0	0	1	1	1	12
13	balance	balance	FM questionnaire	disease-specific questionnaire	0	1	0	0	0	0	1	13
14	bladder	bladder	IC/BPS questionnaire; ANATO	anatomical vocabulary; disease-	1	0	0	0	1	1	1	14

			MY_LEX	specific questi...								
15	bloat	bloated	PAIN_LEX	pain-related vocabulary	0	0	1	0	0	1	0	15
16	body	body	ANATOMY_LEX	anatomical vocabulary	0	0	0	0	1	1	0	16
17	bother	bother	IC/BPS questionnaire	disease-specific questionnaire	1	0	0	0	0	0	1	17
18	bright	bright	FM questionnaire	disease-specific questionnaire	0	1	0	0	0	0	1	18
19	burn	burn	IC/BPS questionnai	disease-specific ques	1	0	1	0	0	1	1	19

			re; PAIN_LEX	tionnaire; pain-related v...								
20	burns	burns	FM questionnaire	disease-specific questionnaire	0	1	0	0	0	0	1	20
21	buttock	buttock	FM questionnaire; ANATOMY_LEX	anatomical vocabulary; disease-specific questi...	0	1	0	0	1	1	1	21
22	cervix	cervix	ANATOMY_LEX	anatomical vocabulary	0	0	0	0	1	1	0	22
23	chest	chest	FM questio	anatomical	0	1	0	0	1	1	1	23

			nnaire; ANATOMY_LEX	vocabulary; disease-specific questi...								
24	chronic	chronic	PAIN_LEX	pain-related vocabulary	0	0	1	0	0	1	0	24
25	cognitive	cognitive	FM questionnaire	disease-specific questionnaire	0	1	0	0	0	0	1	25
26	cold	cold	FM questionnaire	disease-specific questionnaire	0	1	0	0	0	0	1	26
27	concentrate	concentrate	FM questionnaire	disease-specific question	0	1	0	0	0	0	1	27

				naire								
28	cramp	cramp	PAIN_LEX	pain-related vocabulary	0	0	1	0	0	1	0	28

Supplementary Table S5.Exclusion-word list used during preprocessing

Complete list of exclusion words removed before network construction. Excluded terms consisted primarily of function words, questionnaire instructions, administrative text, and other non-informative vocabulary not relevant to symptom-language analysis.

Supplementary Note 2. (Step 2A)

Dense-core topology analysis

Supplementary Table S6. Weighted Louvain statistics of dense-core networks

Weighted Louvain clustering was applied to the dense-core networks extracted from the IC/BPS and FM symptom-language networks. Dense cores were defined using the top 12% of nodes ranked by weighted eigenvector centrality followed by k-core filtering. Network density, average degree, community number, modularity (Q), and resolution parameter (γ) are summarized in Supplementary Table S6.

The FM dense core exhibited complete connectivity (density = 1.000) and an extremely low modularity value (Q = 0.001), indicating minimal community structure despite the presence of weak algorithmic partitions. In contrast, the IC/BPS dense core remained highly interconnected (density = 0.956) while retaining weak residual modular organization (Q = 0.095), consistent with residual substructure within the dense core. These findings support a topological contrast between a fully saturated FM dense core and a partially differentiated IC/BPS dense core.

Supplementary Table S7. Sensitivity analyses of dense-core topology across alternative eigenvector-centrality thresholds and k-core parameters

Sensitivity analyses were performed to evaluate the robustness of dense-core topology under alternative node-selection thresholds and k-core parameters. Eigenvector-centrality thresholds of 10%, 12%, and 15% were examined, together with k-core values of 6, 8, and 10.

Across all threshold settings, the FM dense core remained fully connected or near fully connected, with density values approaching 1.000 and negligible modularity values. In contrast, the IC/BPS network consistently retained non-zero modularity values, indicating persistent residual topological differentiation despite similarly high levels of connectivity. Similar patterns were observed across alternative k-core parameters. These results suggest that the observed

contrast between complete dense-core saturation in FM and residual modular differentiation in IC/BPS is not attributable to a single parameter choice and remains stable across a range of analytical settings.

Network	Nodes	Edges	Density	Avg degree	Modularity	Network	Nodes
IC/BPS	10	43	0.956	8.6	0.095	IC/BPS	10
FM	18	153	1.000	17.0	0.001	FM	18

Supplementary Table S6. Weighted Louvain statistics of dense-core networks

Dense-core topology statistics for interstitial cystitis/bladder pain syndrome (IC/BPS) and fibromyalgia (FM). Dense-core extraction: top 12% weighted eigenvector-centrality nodes followed by k-core filtering. Metrics: node number, edge number, network density, average degree, community number, and modularity (Q). Community assignment: weighted Louvain clustering.

**Part A. Eigenvector-centrality threshold sensitivity**						
Disease	Threshold	Nodes	Edges	Density	Communities	Modularity Q
IC/BPS	Top 10%	8	28	1.000	2	0.048
IC/BPS	Top 12%	10	43	0.956	2	0.095
IC/BPS	Top 15%	12	62	0.939	2	0.101
FM	Top 10%	15	105	1.000	1	0.001
FM	Top 12%	18	153	1.000	1	0.001
FM	Top 15%	22	231	1.000	2	0.003
**Part B. k-core sensitivity analysis after top-12% eigenvector selection**						
Disease	k-core	Nodes	Edges	Density	Communities	Modularity Q
IC/BPS	6	10	43	0.956	2	0.095
IC/BPS	8	9	36	1.000	2	0.054
IC/BPS	10	0	0	NA	0	NA
FM	6	18	153	1.000	1	0.001

FM	8	18	153	1.000	1	0.001
FM	10	18	153	1.000	1	0.001

Supplementary Table S7. Sensitivity analyses of dense-core topology across alternative eigenvector-centrality thresholds and k-core parameters

Part A summarizes dense-core topology after selecting the top 10%, 12%, and 15% of nodes ranked by weighted eigenvector centrality. Part B summarizes k-core sensitivity following top-12% eigenvector-centrality selection using k = 6, 8, and 10. Metrics include node number, edge number, network density, Louvain community number, and modularity (Q). Community assignment was performed using weighted Louvain clustering (γ = 1.0). Across alternative parameter settings, the FM dense core consistently exhibited complete connectivity (density = 1.000) and negligible modularity values, whereas the IC/BPS dense core retained non-zero modularity values despite high density, supporting the robustness of the observed topological contrast.

Supplementary Note 3. (Step 2B)

Supplementary Table S8 shows pain-centered lexical triads after disease-label exclusion. To evaluate the influence of disease-label anchoring, disease-label nodes were removed before triad extraction (IC/BPS: “cystitis”; FM: “fibromyalgia”). Pain-centered lexical triads were then recalculated using the modified dense-core networks. Pain-centered triads remained identifiable in both conditions after disease-label exclusion. The number of pain-centered triads decreased from 34 to 26 in IC/BPS and from 136 to 120 in FM. These findings indicate that pain-centered triadic motifs were not solely dependent on disease-label nodes and persisted after removal of the dominant diagnostic terms.

Supplementary Table S9 shows the final node composition of the dense-core networks. Dense-core node composition was examined to improve transparency of the dense-core extraction procedure. Nodes retained in the final dense-core networks were ranked according to weighted eigenvector centrality. The IC/BPS dense core consisted of 10 nodes, whereas the FM dense core consisted of 18 nodes. These node lists represent the final dense-core networks used for weighted Louvain clustering and pain-centered triad analyses.

Supplementary Table S10 shows dense-core extraction outputs under alternative analytical settings. Sensitivity analyses were performed to evaluate the robustness of dense-core topology across alternative weighted eigenvector-centrality thresholds and k-core parameters. Dense-core topology statistics were calculated using weighted Louvain clustering with a fixed resolution parameter ($\gamma = 1.0$). Across alternative extraction settings, the FM dense core remained fully connected or near fully connected with negligible modularity values, whereas the IC/BPS dense core retained non-zero modularity values. These findings support the stability of the observed topological contrast between the two conditions.

Condition	Rank	Core triad	Triangle share	

		("pain"–a–b)	score	
0	IC/BPS	1	"pain"–"back"–"pelvic"	0.083
1	IC/BPS	2	"pain"–"pelvic"–"cold"	0.072
2	IC/BPS	3	"pain"–"back"–"cold"	0.062
3	IC/BPS	4	"pain"–"pelvic"–"test"	0.061
4	IC/BPS	5	"pain"–"back"–"test"	0.056
5	IC/BPS	6	"pain"–"pelvic"–"body"	0.053
6	IC/BPS	7	"pain"–"pelvic"–"sleep"	0.050
7	IC/BPS	8	"pain"–"pelvic"–"avoid"	0.048
8	IC/BPS	9	"pain"–"back"–"body"	0.048
9	IC/BPS	10	"pain"–"back"–"avoid"	0.044
10	FM	1	"pain"–"sleep"–"body"	0.024
11	FM	2	"pain"–"fatigue"–"sleep"	0.018
12	FM	3	"pain"–"sleep"–"flare"	0.018
13	FM	4	"pain"–"sleep"–"stress"	0.017
14	FM	5	"pain"–"chronic"–"sleep"	0.017
15	FM	6	"pain"–"sleep"–"muscle"	0.017
16	FM	7	"pain"–"flare"–	0.016

			"body"	
17	FM	8	"pain"–"fatigue"–"body"	0.016
18	FM	9	"pain"–"sleep"–"hurt"	0.015

Supplementary Table S8. Pain-centered lexical triads after disease-label exclusion

Pain-centered lexical triads ("pain"–a–b) after removal of disease-label nodes from each dense-core network. Disease-label exclusion: "cystitis" (IC/BPS) and "fibromyalgia" (FM). Triangle share score: relative contribution of each triad to all pain-centered triangles within the corresponding dense core.

	Condition	Rank by eigenvector	Dense-core node
0	IC/BPS	1	pain
1	IC/BPS	2	cystitis
2	IC/BPS	3	pelvic
3	IC/BPS	4	back
4	IC/BPS	5	test
5	IC/BPS	6	cold
6	IC/BPS	7	body
7	IC/BPS	8	avoid
8	IC/BPS	9	shame
9	IC/BPS	10	sleep
10	FM	1	pain
11	FM	2	fibromyalgia
12	FM	3	sleep
13	FM	4	body
14	FM	5	flare
15	FM	6	fatigue
16	FM	7	chronic
17	FM	8	stress
18	FM	9	muscle

19	FM	10	test
20	FM	11	hurt
21	FM	12	cold
22	FM	13	severe
23	FM	14	nerve
24	FM	15	anxiety
25	FM	16	frustrat
26	FM	17	nervous

Supplementary Table S9. Dense-core node composition

Final node membership of the IC/BPS and FM dense-core networks after weighted eigenvector-centrality filtering and dense-core extraction. Node ranking according to weighted eigenvector centrality.

Condition	Analysis type	Extraction pipeline	Eigenvector-centrality threshold	k-core parameter	Final dense-core nodes	Final dense-core edges	Density	Average degree	Weighted Louvain communities	Weighted modularity Q	Resolution parameter gamma	
0	IC/BPS	main_top12	top_eigenvector_only	0.12	NaN	10	43	0.956	8.600	2	0.095	1.0
1	FM	main_top12	top_eigenvector_only	0.12	NaN	18	153	1.000	17.000	1	0.000	1.0
2	IC/BPS	top_eigenvector_threshold	top_eigenvector_only	0.10	NaN	8	28	1.000	7.000	2	0.048	1.0
3	FM	top_eigen	top_eigen	0.1	N	15	10	1.0	14.0	1	0.0	1

		vector_threshold	vector_only	0	aN		5	00	00		00	.0
4	IC/BPS	top_eigenvector_threshold	top_eigenvector_only	0.12	NaN	10	43	0.956	8.600	2	0.095	1.0
5	FM	top_eigenvector_threshold	top_eigenvector_only	0.12	NaN	18	153	1.000	17.000	1	0.000	1.0
6	IC/BPS	top_eigenvector_threshold	top_eigenvector_only	0.15	NaN	12	62	0.939	10.333	2	0.101	1.0
7	FM	top_eigenvector_threshold	top_eigenvector_only	0.15	NaN	22	231	1.000	21.000	2	0.003	1.0
8	IC/BPS	k_core_after_top12	top_eigenvector_plus_kcore	0.12	6.0	10	43	0.956	8.600	2	0.095	1.0
9	FM	k_core_after_top12	top_eigenvector_plus_kcore	0.12	6.0	18	153	1.000	17.000	1	0.000	1.0
10	IC/BPS	k_core_after_top12	top_eigenvector_plus_kcore	0.12	8.0	9	36	1.000	8.000	2	0.054	1.0
11	FM	k_core_after_top12	top_eigenvector_plus_kcore	0.12	8.0	18	153	1.000	17.000	1	0.000	1.0
12	IC/BPS	k_core_after_top12	top_eigenvector_plus_kcore	0.12	10.0	0	0	0.000	0.000	0	NaN	1.0

Supplementary Table S10. Dense-core extraction workflow and sensitivity analysis

Dense-core topology statistics across alternative weighted eigenvector-centrality thresholds and k-core parameters. Metrics: node number, edge number, network density, average degree, weighted Louvain community number, modularity (Q), and resolution parameter (γ).

Supplementary Note 4. (Step 3)

Supplementary Table S11 shows first-order lexical neighbors of “pain” after strict exclusion of disease-label terms, generic discourse terms, and ambiguous terms. To evaluate the influence of disease-label anchoring and non-specific discourse terms, first-order neighbors of “pain” were recalculated after exclusion of disease-label nodes (“cystitis”, “interstitial”, and related IC/BPS terms; “fibromyalgia” and related FM terms), generic discourse terms, and reviewer-identified ambiguous terms. Disease-specific enrichment was recalculated using corpus-size-normalized rates per million words and log2 rate ratios. FM-enriched neighbors included fatigue, chronic, stress, muscle, nerve, depression, and nervous, whereas IC/BPS-enriched neighbors included pelvic, urethra, kidney, vaginal, urinate, and bloated. Similar directional patterns remained identifiable after exclusion of disease-label and non-specific terms. These findings indicate that differential pain-centered lexical neighborhoods were not solely dependent on dominant diagnostic labels or generic discourse terms.

direction	term	display_term	w_FM	w_IC	rate_FM_per_million	rate_IC_per_million	delta_rate_per_million	log2_rate_ratio_FM_vs_IC	
0	FM-enriched	fatigue	fatigue	1393	0	1297.08	0.00	1297.08	11.342
1	FM-enriched	chronic	chronic	1257	0	1170.44	0.00	1170.44	11.193
2	FM-enriched	stress	stress	1184	0	1102.47	0.00	1102.47	11.107

3	FM-enriched	muscle	muscle	1140	0	1061.50	0.00	1061.50	11.053
4	FM-enriched	hurt	hurt	905	0	842.68	0.00	842.68	10.720
5	FM-enriched	nerve	nerve	801	0	745.84	0.00	745.84	10.544
6	FM-enriched	severe	severe	674	0	627.59	0.00	627.59	10.295
7	FM-enriched	depression	depression	617	0	574.51	0.00	574.51	10.167
8	FM-enriched	nervous	nervous	607	0	565.20	0.00	565.20	10.144
9	FM-enriched	frustrat	frustrated	494	0	459.98	0.00	459.98	9.847
10	IC/BPS-enriched	back	back	0	1740	0.00	3311.35	-3311.35	-12.693
11	IC/BPS-enri	joint	joint	0	82	0.00	156.05	-156.05	-8.291

	ched								
12	IC/BPS-enriched	vagina	vagina	0	65	0.00	123.70	-123.70	-7.957
13	IC/BPS-enriched	penis	penis	0	29	0.00	55.19	-55.19	-6.799
14	IC/BPS-enriched	vaginal	vaginal	17	238	15.83	452.93	-437.10	-4.795
15	IC/BPS-enriched	urinate	urinate	0	6	0.00	11.42	-11.42	-4.575
16	IC/BPS-enriched	pelvic	pelvic	205	2198	190.88	4182.96	-3992.08	-4.450
17	IC/BPS-enriched	urethra	urethra	5	32	4.66	60.90	-56.24	-3.574
18	IC/BPS-enriche	kidney	kidney	68	275	63.32	523.35	-460.03	-3.037

	d								

Supplementary Table S11. Strictly filtered first-order lexical neighbors of "pain"

First-order lexical neighbors of "pain" after exclusion of disease-label terms, generic discourse terms, and ambiguous terms. Disease-label exclusion: "cystitis", "interstitial", and related IC/BPS terms; "fibromyalgia" and related FM terms. Additional exclusion: generic discourse terms and reviewer-identified ambiguous terms. Disease enrichment by corpus-size-normalized rates per million words and log2 rate ratios. Positive log2 values: FM-enriched co-occurrence with "pain". Negative log2 values: IC/BPS-enriched co-occurrence with "pain".
